# High Estrogen Levels Cause Greater Leg Muscle Fatigability in Eumenorrheic Young Women after 4 mA Transcranial Direct Current Stimulation

**DOI:** 10.3390/brainsci12040506

**Published:** 2022-04-15

**Authors:** Justin R. Deters, Alexandra C. Fietsam, Craig D. Workman, Thorsten Rudroff

**Affiliations:** 1 Department of Health and Human Physiology, University of Iowa, Iowa City, IA 52242, USA; justin-deters@uiowa.edu (J.R.D.); alexandra-fietsam@uiowa.edu (A.C.F.); craig-workman@uiowa.edu (C.D.W.); 2 Department of Neurology, University of Iowa Hospitals and Clinics, Iowa City, IA 52242, USA

**Keywords:** tDCS, menstrual cycle, estrogen, fatigue, electromyography

## Abstract

Transcranial direct current stimulation (tDCS) research has shown great outcome variability in motor performance tasks, with one possible source being sex differences. The goal of this study was to evaluate the effects of estrogen levels on leg muscle fatigability during a fatigue task (FT) after 4 mA tDCS over the left motor cortex (M1). Ten young, healthy eumenorrheic women received 4 mA anodal active or sham stimulation over the left M1 during periods of high and low estrogen levels. A fatigue index (FI) was calculated to quantify fatigability, and the electromyography (EMG) of the knee extensors and flexors was recorded during the FT. The findings showed that tDCS applied during high estrogen levels resulted in greater leg muscle fatigability. Furthermore, a significant increase in EMG activity of the right knee extensors was observed during periods of active stimulation, independent of estrogen level. These results suggest that estrogen levels should be considered in tDCS studies with young healthy women.

## 1. Introduction

Hormonal differences between women and men have been shown to independently affect brain stimulation-induced changes in cortical excitability [1,2,3], and previous studies have investigated these effects using repetitive transcranial magnetic stimulation (rTMS) [4,5]. Furthermore, a multivariate meta-regression of transcranial direct current stimulation (tDCS) targeting the frontal brain regions [6] revealed that biological sex moderated the effects of the applied stimulation dose (i.e., current density and density charge) on cognitive outcomes; specifically, a higher proportion of females in a given study led to larger effect sizes that significantly contributed to the regression model [6]. The authors suggested a few reasons for this finding and noted sex hormones, which can modulate endogenous cortical excitability, as the most plausible explanation. However, most tDCS studies have not considered sex differences as an outcome-modifying factor [7]. This neglect might be slowing advances in the tDCS field, and could partially explain the high inconsistency rates [8,9,10,11,12,13,14] and inter-subject variability (e.g., approximately 50% of subjects might not respond to tDCS [15]) common in tDCS investigations.

Increased levels of estrogen, one of the two primary female sex hormones, has been associated with increases in cortical excitability [4,5,16], which suggests that estrogen may reinforce endogenous excitatory mechanisms in the cortex. However, because anodal tDCS also enhances cortical excitability when targeted [17,18], excessive excitability [19] when estrogen levels are high is also possible. In theory, one possible product of anodal tDCS application while estrogen levels are high is cortical overexcitation in the target area. This momentary disruption of neurophysiological homeostasis might result in non-optimal or decreased motor performance. This is consistent with recent findings that anodal M1 tDCS resulted in increased leg muscle fatigability in young women when compared to young men [20]; sex-specific biological variations (e.g., skull characteristics, sex hormones) were speculated to have caused these findings. Specifically, modeling studies have reported that sex-specific differences in skull characteristics, which are different between women and men, modulate the amount of current that reaches the cortex [21,22]. However, no study has examined if and how fluctuating estrogen levels alter the effects of tDCS on human performance (e.g., fatigue outcomes).

Fatigue has been defined as “the decrease in physical and/or mental performance that results from changes in central, psychological, and/or peripheral factors” [23] and is commonly examined in tDCS studies. Specifically, performance fatigability might be influenced by changes in corticospinal excitability [24] and/or alterations in motor unit recruitment strategies [25], either of which may be altered by tDCS. However, the results of such studies are conflicting. Some have reported reduced performance fatigability from tDCS (see [26] for a review), while others have found that tDCS increases muscle fatigability, especially in young, healthy subjects [19,20,27]. Given the potential for hormones (specifically estrogen) to modulate tDCS efficacy, there is a surprising lack of knowledge regarding how estrogen might affect tDCS performance outcomes (e.g., leg muscle fatigability) in healthy young women.

Therefore, research assessing the influence of sex hormones on stimulation-related functional outcomes is vital to fully understand the underlying mechanisms of tDCS and maximize test results. The purpose of this study was to examine the influence of endogenous estrogen levels on leg muscle fatigability after 4 mA tDCS in eumenorrheic young women. It was hypothesized that high estrogen levels would result in increased leg muscle fatigability in active tDCS compared to sham, based upon our previous work [20] and the known effects of estrogen in the cortex [5,16]. This work is vital because determining the influence of fluctuating hormones in women on stimulation outcomes may be an especially important factor to reduce response variability in tDCS research.

## 2. Materials and Methods

### 2.1. Subjects

A power analysis was performed based on our previous study (η^2^ = 0.18) [20], and it was found that 8 subjects were needed to achieve 80% power at α = 0.05. Thus, to account for 20% attrition, a total of 10 healthy, tDCS-naïve young eumenorrheic women were recruited for this study. The inclusion criteria were: (1) biological woman between the ages of 18 and 35; (2) right-side dominant (as brain morphology differences may exist between right- and left-side-dominant people [28]); (3) physically active (had performed at least 30 min of moderate-intensity physical activity on 3 or more days of the week, during the last 3 months) [29]; (4) without other chronic medical conditions (e.g., neurological or psychiatric); and (5) not using psychoactive medications. The exclusion criteria included: (1) pregnancy or currently trying to become pregnant; (2) hormonal contraceptives/supplement use; (3) no contraindications to tDCS based on previous studies (e.g., fissures, holes, or implanted devices in the skull [30]). The study was performed in accordance with the Declaration of Helsinki and was approved by the Institutional Review Board at the University of Iowa. All subjects provided written consent before participation.

### 2.2. Study Design

This study employed a double-blind, randomized, crossover design, wherein all subjects received both active and sham stimulation during each phase of their menstrual cycle. Each of the 10 subjects completed a total of 6 visits to the lab. The first visit was a familiarization visit where maximal strength testing was performed to confirm leg dominance, and an isokinetic fatigue task (FT) was completed to familiarize each subject with the FT performed during the evaluation sessions. Additionally, subjects were asked about the start of their previous menstrual cycle, along with the projected start of their next menstrual cycle. The start of the menstrual cycle was deemed the first day of menstruation. Sessions were then scheduled to correspond with high and low points of estrogen level throughout the cycle. The early follicular phase (days 3–4) was targeted for low estrogen levels. The late follicular phase (days 9–10) and mid-luteal phase (days 18–20) were targeted for high estrogen levels. Sessions were scheduled so that each subject had both active (4 mA) and sham tDCS during each phase (early follicular, late follicular, and mid-luteal) of their menstrual cycle to fully assess the effects of stimulation. Due to the diurnal nature of estrogen, visits 2–6 were scheduled at the same time of day for each individual subject (i.e., ±2 h for each session) [31]. Testing sessions began with a blood draw and assay to assess serum estrogen levels, followed by tDCS administration (active or sham), and right and left leg FTs.

### 2.3. Isokinetic/Isometric Strength Testing

Strength testing was performed on a HUMAC NORM isokinetic dynamometer (CSMi, Stoughton, MA, USA). Before beginning the strength testing, subjects completed a warm-up exercise which consisted of 15 repetitions of knee extension and knee flexion (60°/s, concentric/concentric). After resting for ≥30 s, three repetitions of maximal strength isometric knee extension and flexion were completed at 65° and 30°, respectively, with ≥30 s of rest between each repetition. Then, five repetitions of maximal strength isokinetic knee extension and flexion were completed at 60°/s (concentric/concentric) with ≥30 s of rest between each set. The right leg was always tested first, followed by the left. Strong verbal encouragement was provided during each repetition to ensure maximal effort from the participants. The greatest torque value acquired during either of the strength testing tasks (isometric or isokinetic) was utilized to objectively confirm right-side dominance.

### 2.4. Isokinetic Fatigue Task (FT)

This FT protocol was identical to protocols assessing the effects of tDCS on fatigability previously performed in this lab [19,20,32]. The FT comprised 40 consecutive repetitions of maximal effort isokinetic knee extension and flexion (120°/s, concentric/concentric) [27,33]. In visits 2–5, a 15-repetition warm-up exercise was completed before the FT, as detailed above. The FT was performed on the dominant (right) leg first and ≥5 min rest was provided to allow for adequate heart rate and respiratory recovery before starting the left leg FT. Vigorous verbal encouragement and visual feedback (i.e., per rep work bars) were given to ensure the participants were performing maximal effort contractions throughout the fatigue tasks. The largest torque from each repetition was included in the analysis.

### 2.5. Electromyography (EMG)

Muscle activity throughout the strength and fatigue tasks was recorded via a wireless EMG system (Ultium-EMG, Noraxon, USA Inc., Scottsdale, AZ, USA). EMG electrodes (3M Red Dot Monitoring Electrode, Model 2560; 3M Corp., St. Paul, MN, USA; 2 cm between each 1.3 cm effective area) were secured bilaterally over the rectus femoris, vastus medialis, vastus lateralis, and semitendinosus corresponding to a 3D muscle map which followed SENIAM guidelines, and was provided by the EMG software (MR 3.14, myoMUSCLE, Noraxon USA Inc., Scottsdale, AZ, USA). To increase electrode placement consistency, the same researchers placed the electrodes on the subjects during each visit. The electrode sites were prepared by shaving and cleaning the electrode site with an alcohol wipe before placing the electrodes. The electrodes and wireless transmitters were secured in place with elastic bandages and EMG data were collected at 2000 Hz.

### 2.6. Transcranial Direct Current Stimulation (tDCS)

A tDCS device (Soterix Medical Inc, New York, NY, USA) delivered a small direct current to the scalp through two carbon electrodes which were situated inside two saline-soaked 5 cm × 7 cm sponge electrodes (area = 35 cm^2^; EASYpad, Soterix Medical Inc., New York, NY, USA). The anode was secured over the left M1 (C3 according to the 10–20 EEG convention) to stimulate the motor cortex representation of the dominant (right) leg, and the cathode was secured over the contralateral (left) supraorbital area (just inferior to Fp2). The medial edge of the anode always abutted or slightly covered Cz, which ensured that this electrode was unilaterally targeting the M1 area in the longitudinal fissure that represents the leg [34]. The sponge electrodes were secured by an EASYstrap (Soterix Medical Inc, New York, NY, USA), which had ruler-like markings to allow for consistent electrode placement between sessions. Previous work in our lab has demonstrated increased fatigability in healthy subjects after 2 and 4 mA active stimulation over the motor cortex compared to sham [19]. Further analysis of this dataset revealed sex-specific differences in the FT when subjects were further stratified by biological sex. Women were more fatigued than men after 4 mA tDCS, but not after 2 mA [20]. Therefore, because we observed sex differences when using 4 mA only, and not 2 mA stimulation, 4 mA (current density = 0.11 mA/cm^2^) was used as the stimulation intensity in this study to further elucidate the effects of estrogen on fatigability. Active stimulation began with a 30 s ramp-up, was held constant at 4 mA for 20 min, and then slowly ramped back down to 0 mA over 30 s. During sham stimulation, the device ramped up to 4 mA over 30 s, then promptly ramped down to 0 mA over 30 s. The stimulation intensity was then held at 0 mA for 20 min before the ramp-up/down process was repeated. After stimulation, the subjects sat quietly for 10 min to provide adequate time for the stimulation effects to peak [35,36] before beginning the FT. tDCS tolerability was evaluated by asking subjects to describe the sensations they experienced and to rate those sensations on a 10-point Likert scale (1 = “barely perceptible”, 10 = “worst sensation I could possibly stand” [37]. The subjects were informed that they would randomly receive either sham or active stimulation during each of the five experimental sessions. After each session, they were asked what stimulation condition they thought they received and to determine their confidence in their guess on a 10-point Likert scale (1 = “not confident at all”; 10 = “extremely confident” [37]. The same researcher scheduled participants according to their menstrual cycle phase and administered tDCS. The subjects and other study personnel were blinded to the stimulation conditions of each testing session until after the final session of each subject was completed.

### 2.7. Blood Draw

Sessions 2–6 began with a blood draw in the Clinical Research Unit at the University of Iowa Hospital and Clinics (UIHC). Staff nurses collected 4.5 mL blood from the median cubital vein of the left arm (total volume collected per subject = 9 mL) for the estrogen assay. Samples were immediately analyzed for serum estrogen levels after the blood draws by UIHC Pathology technicians using an Electrochemiluminescence Assay (Roche Diagnostics, Basel, Switzerland). The estrogen assay had a lower limit of detection of 5 pg/mL and a coefficient of variation of 8%. Because menstrual cycles have great inter-and intrasubject variability, and current menstrual cycle evaluation methods are inefficient [38], the peak estrogen levels of the subjects were not consistently found in the late-follicular phase, which is a common failing of menstrual cycle phase calendar estimation [39]. Thus, estrogen levels were grouped as high or low according to each individual subject’s estrogen serum levels, irrespective of the anticipated/targeted phase. This ranged from 22–432 pg/mL for high values and 9–183 pg/mL for low values. For example, subject 8 had a high value of 432 pg/mL and a low value of 44, while subject 4 had a high value of 168 pg/mL and a low value of 39 pg/mL.

### 2.8. Data Analysis

A torque-derived fatigue index (FI-T) was computed to assess the effect of tDCS and sex hormones on leg muscle fatiguability. The FI-T was calculated using the greatest torque from the relevant repetitions of the FT as follows: ([mean of reps 3 through 7—mean of last five reps]/mean of reps 3 through 7) × 100 [33,40,41]. Higher FI-T values indicate increased muscle fatigability (i.e., a greater difference in torque production between the beginning and the end of the FT) and were interpreted as a poorer FT performance. The EMG interference signals from each muscle were bandpass filtered (3.5 Hz–350 Hz) [27,32,42,43], rectified, and smoothed (50 ms root mean square window). Peak EMG activity from the strength testing was used as a normalization value for EMG during the FT. The muscle activity of the knee extensors (rectus femoris, vastus medialis, and vastus lateralis) was averaged to represent the cumulative activity of this muscle group. The first two repetitions of the FT were considered adaptation repetitions and were removed. Therefore, the remaining 38 repetitions were used for the FI-T and average EMG (aEMG) analyses [19,20,33]. The subsequent 38 repetitions were organized into 8 windows. The first seven windows consisted of five consecutive and non-overlapping repetitions (e.g., window 2 = reps 8–12; window 3 = reps 13–17, etc.) while the last (eighth) window was comprised of the final three repetitions [33]. All EMG data were analyzed in the myoMUSCLE software (MR3 Version 3, Noraxon USA Inc., Scottsdale, AZ, USA) and torque data were calculated within and exported from the HUMAC2015 software (CSMI, Stoughton, MA, USA).

### 2.9. Statistical Analysis

A stimulation condition (active vs. sham) by estrogen level (high vs. low) repeated measures ANOVA was performed on the FI values and EMG activity for the right and left extensors and flexors. Post hoc analyses (paired *t*-tests and Bonferroni correction) and effect size (Cohen’s d) were calculated to clarify significant main effects and interactions. Significance was accepted at *p* ≤ 0.05. Normality and sphericity assumptions were evaluated with the Shapiro–Wilk test and Mauchly’s test of sphericity for the ANOVAs. Greenhouse–Geisser corrections were used when the sphericity assumption was violated. GraphPad Prism 9 (GraphPad Software, San Diego, CA, USA) was utilized to perform the analyses.

## 3. Results

### 3.1. Subject Characteristics

All subjects (*n* = 10; age = 24.3 ± 5.5 years; height = 164.1 ± 6.5 cm; weight = 61.5 ± 10.8 kg) completed all study visits. Data are reported as mean ± SD in the text and mean ± SEM in the figures. All normality and sphericity assumptions for ANOVAs and paired *t*-tests were evaluated a priori and were sufficiently met.

### 3.2. Muscle Fatigability

**Figure 1:**
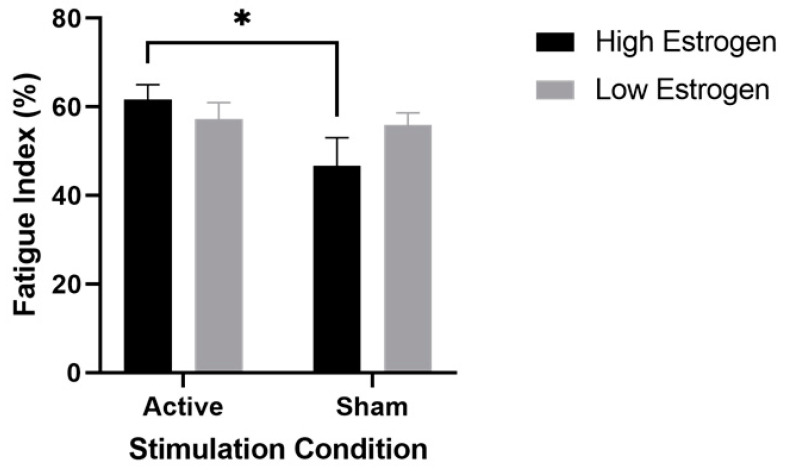
Fatigue index (FI) of the right extensors, stratified by tDCS condition and estrogen level. Data are mean ± SEM. * represents a significant increase (*p* = 0.04) in the active stimulation vs. the sham condition when estrogen was high.

**Figure 2:**
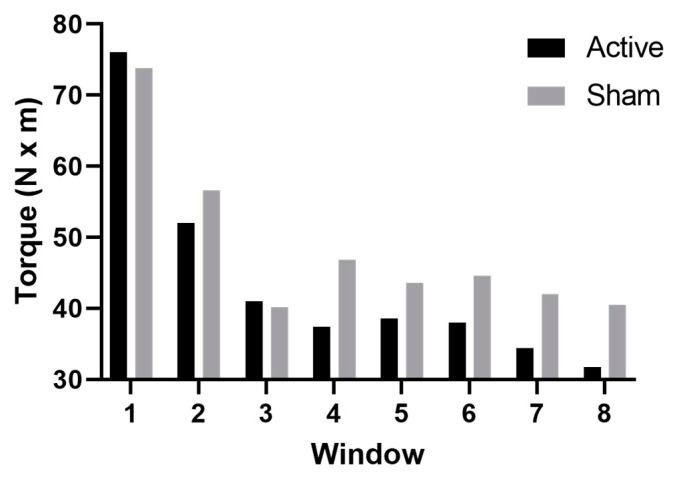
Average torque during the FT divided into 8 time windows in a representative subject during active (black) and sham (grey) stimulation when estrogen levels were high. The bars represent the average torque calculated for each time window.

The results of the ANOVAs indicated a significant main effect of stimulation condition for the right extensors (F (1,9) = 8.2, *p* = 0.02, η^2^ = 0.46) and a significant estrogen level x stimulation condition interaction for both the right (F (1,9) = 5.2, *p* = 0.05, η^2^ = 0.37) and left (F (1,9) = 10, *p* = 0.01, η^2^ = 0.53) extensors. Figure 1 displays the results of the post hoc testing for the right extensors which revealed that FI was significantly higher (i.e., greater fatigability) in the high-estrogen active condition compared to the high-estrogen sham condition (*p* = 0.04, d = 0.94; 61.7 ± 10.6 vs. 46.7 ± 20.0). On the other hand, the difference between low-estrogen active and low-estrogen sham was not significant (*p* > 0.99, d = 0.13, 57.28 ± 11.57 vs. 55.90 ± 8.67), indicating that only the combination of high estrogen and active tDCS altered leg muscle fatigability. Post hoc testing for the left extensors revealed similar results, with high FIs after active stimulation showing a similar pattern to sham during high estrogen levels (*p* = 0.02, d = 1.05; 59.6 ± 8.4 vs. 50.9 ± 7.6), with only the combination of high estrogen and active tDCS altering leg muscle fatigability. Similar to the right extensors, the difference between low-estrogen active and low-estrogen sham in the left extensors was not significant (*p* > 0.99, d = 0.12, 54.25 ± 13.99 vs. 55.78 ± 11.42). Figure 2 displays the change in torque production of the right extensors over the eight time windows of a representative subject during high estrogen levels, and highlights the decreased torque production after active stimulation compared to sham. All other right and left extensor post hoc comparisons were nonsignificant (0.99 ≤ *p* ≤ 0.2). Similarly, the results of the ANOVAs for the FI of the right and left flexors revealed no significant main effect of stimulation (right flexors: F (1,9) = 0.92, *p* = 0.36; left flexors: F (1,9) = 0.16, *p* = 0.70) or estrogen level (right flexors: F (1,9) = 1.5, *p* = 0.26; left flexors: F (1,9) = 0.65, *p* = 0.44), and no significant stimulation x estrogen level interaction (right flexors: F (1,9) = 1.7, *p* = 0.23; left flexors: F (1,9) = 0.42, *p* = 0.54).

### 3.3. Muscle Activity

**Figure 3:**
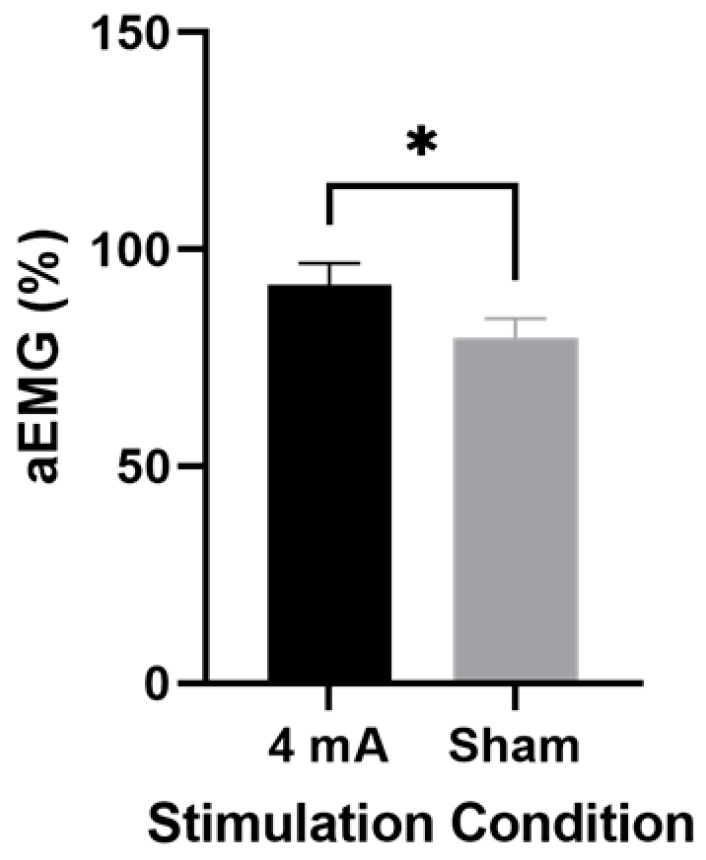
Average EMG values of the right extensors stratified by tDCS condition regardless of estrogen level. Data are mean ± SEM. * indicates that active stimulation is significantly greater (*p* = 0.04) than sham stimulation.

The ANOVA for EMG activity of the right extensors showed a significant main effect of stimulation condition (F (1,9) = 7.4, *p* = 0.02, η^2^ = 0.28), but no significant main effect of estrogen level (F (1,9) = 0.04, *p* = 0.85) or interaction (F (1,9) = 0.75, *p* = 0.41). Figure 3 depicts the results of the post hoc testing for the right extensors, which showed significantly higher average EMG activity during active stimulation compared to sham, regardless of estrogen level (91.8 ± 22.0 vs. 79.7 ± 19.0; *p* = 0.04, d = 0.59). The ANOVAs for the EMG activity of the left extensors and right and left knee flexors revealed no significant main effects of stimulation (F (1,9) ≤ 1.0; *p* ≥ 0.34) or estrogen level (F (1,9) ≤ 2.1; *p* ≥ 0.18) and no interaction (F (1,9) ≤ 2.4; *p* ≥ 0.17).

### 3.4. tDCS Tolerability and Blinding

Tolerability results are summarized below in Table 1. For stimulation blinding, 56.3% correctly guessed that they had received active stimulation, with a confidence of 6.44 ± 1.8.

## 4. Discussion

The purpose of this study was to evaluate the effects of estrogen level on a previously established leg muscle fatigability response to M1 anodal tDCS [19,20]. The findings revealed that only tDCS applied during high estrogen levels resulted in greater leg muscle fatigability in eumenorrheic young women. Furthermore, a significant increase in the EMG activity of the right knee extensors was observed during active stimulation, independent of estrogen level.

### 4.1. High Estrogen and Active Stimulation in Performance Fatigability

Estrogen affects the brain by rapidly increasing the response of cortical neurons to glutamate [44], which is the primary excitatory neurotransmitter in the cortex [45]. Additionally, high levels of estrogen in the cortex leads to the increased activity of sodium channels and the recruitment of excitatory interneurons [25]. This is significant because anodal tDCS has also been shown to independently increase cortical excitability [24]. However, a recent review by Choi and colleagues [46] concluded that anodal-tDCS-induced cortical excitation may occur via a decrease in γ-aminobutyric acid (GABA) concentration (the primary inhibitory cortical neurotransmitter), rather than an increase in glutamate levels. Thus, estrogen and tDCS might increase excitability via different mechanisms.

Interestingly, only two other studies have examined the effects of estrogen levels (verified with serum measurements) on responses to tDCS [47] and excitatory repetitive transcranial magnetic stimulation (rTMS) [48]. Both investigations found an increased response to stimulation in women with high estrogen compared to women with low estrogen and men. Furthermore, Rudroff and colleagues [7] postulated that administering tDCS during periods of high estrogen levels may lead to cortical overexcitation and poorer fatigue performance (e.g., increased fatigability) in healthy, young subjects. If both tDCS and estrogen increase cortical excitability (via different pathways), the combination of the two might be additive and result in cortical overexcitation. The outcomes of the present study, along with a previous study [20], support this notion and may represent a possible explanation for the increases in muscle fatigability after anodal tDCS found in both studies; however, more direct measures are required to confirm this hypothesis. Nevertheless, overexcitation may be an issue unique to healthy subjects because their excitability is likely already optimized. Therefore, any external induction of excitation (e.g., tDCS, rTMS) in young, healthy female subjects might more easily result in overexcitation and hinder performance. This might be contrasted in neuropathological populations that are common targets for tDCS treatments, such as multiple sclerosis or Parkinson’s disease, because these populations tend to exhibit unique patterns of cortical excitation [49,50,51,52], and the hypothetical risk of overexcitation may be different in these subjects. For example, in a longitudinal study of patients with progressive MS, Ayache et al. [52] found that disability progression was significantly associated with a decline in cortical excitability, as measured by TMS motor-evoked potentials. Furthermore, Caramia et al. [51] suggested that the inflammation and acute demyelination common during MS relapse may lead to decreased M1 neuronal excitability. The resulting decreases in cortical excitability may be a harmful effect from the local inflammatory environment, as it has been demonstrated that various inflammatory cytokines (e.g., interleukins) can affect neuronal function and cortical excitability in MS. Thus, 4 mA tDCS in subjects with decreased cortical excitability at baseline might be beneficial rather than deleterious for MS patients and other neurological populations [53].

### 4.2. Muscle Activity in Active Stimulation vs. Sham

EMG activity was greater during active stimulation compared to sham, independent of estrogen levels, indicating that EMG activity was influenced by tDCS but not estrogen levels. This is contradictory to several studies stating that estrogen influences neuromuscular function [54,55,56,57]. Ansdell et al. [57] compared voluntary activation in eumenorrheic women and women on oral contraceptives during different stages of the menstrual cycle, and found the highest level of voluntary activation (TMS superimposed twitch) in eumenorrheic women when estrogen was highest. However, in the current study, the effects of high levels of estrogen on neuromuscular properties during the menstrual cycle appeared to be negligible. This agrees with several other studies and a recent review that all found no effects of fluctuating hormones throughout the menstrual cycle on muscle activity and fatigability [38,58,59]. Importantly, the review by Janse de Jonge et al. [38] theorized that the absence of effects of fluctuating hormone levels may be due to inconsistent menstrual cycle evaluation methods across the literature. Thus, administering 4 mA active stimulation might result in increased neural drive and/or altered motor unit recruitment strategies independent of estrogen level, which may subsequently increase the EMG activity of the muscles, as has been shown previously in our lab [27].

### 4.3. Crossover Effect

Interestingly, the results also showed effects on the fatigability and EMG activity of the left knee extensors. Our previous study [33] and results from others [60,61] suggest that tDCS over the dominant hemisphere may also influence motor performances originating from the non-dominant hemisphere. Specifically, Mondini et al. [60] demonstrated alterations in spectral EEG power on the side contralateral to stimulation after tDCS, and Park et al. [61] showed the diffuse effects of tDCS on the right hemisphere areas after left dorsolateral prefrontal cortex stimulation. Therefore, the findings of the current study are in line with the notion that tDCS may increase interhemispheric cooperation, particularly in the primary motor cortices, while performing motor tasks [62]. Moreover, it is suggested that there may be a ceiling effect of stimulation in the targeted motor cortex, which might increase fatigability in the ipsilateral limb and result in greater transcallosal inhibition [33].

### 4.4. Limitations and Future Studies

This study has several limitations to consider. The sample size was relatively small (*n* = 10); however, our study was sufficiently powered to find differences based on results from previous works [20]. Future studies with larger samples are recommended to confirm these findings. Future protocols should also evaluate the levels of other hormones (e.g., progesterone, follicular-stimulating hormones, and luteinizing hormones) besides estrogen over multiple cycles to assess the effects of each hormone on tDCS outcomes after both active and sham stimulation. Furthermore, studying menstrual cycles is an inherently challenging task, with large variability in “normal” cycle lengths and hormone levels between women (i.e., what is considered high estrogen for one subject may not be high for another subject). Healthy values for estrogen in eumenorrheic women can vary from 12–400; therefore, there is a wide range of acceptable values. The current study did not follow subjects over multiple cycles, and instead relied on self-reported menses to mark the start of a cycle and the subsequent estrogen peak estimations. Self-reporting led to inconsistencies in estrogen level and cycle phase at the time of assessment; therefore, it was deemed that we did not hit the correct estrogen peaks for some of the subjects. Thus, phases were ignored, and estrogen was grouped into high or low to increase the validity of comparison between subjects. Additionally, recent evidence suggests a high prevalence of anovulatory cycles and luteal phase deficiency in physically active females, with an occurrence of 30% in physically active women, rising to 50% in women exercising ≥ 450 min/week [38,56]. Thus, only including moderately physically active women may have led to irregularities within the menstrual cycles of some of our subjects. Future studies should account for these variations and confirm ovulation via the luteinizing hormone surge (e.g., with a urine test) concurrent with ovulation, or track individual menstrual cycles (and serum hormonal fluctuations) over a longer interval. Moreover, the stimulation in this study was performed with an intensity of 4 mA, while a majority of tDCS studies utilize intensities ≤ 2 mA [26,63,64,65,66]. It has been suggested that women might receive significantly less current at the cortex from tDCS than men when stimulation is applied with the same parameters [21,67]. Thus, prospective investigations should include both 2 mA and 4 mA intensities to determine if the hypothesized estrogen-induced overexcitation is dose-dependent, or if it only occurs at a higher intensity. Lastly, it is also possible that iron concentrations play a role in fatigue [59]. Indeed, as many as 30% of female college athletes have low iron [68], which could be a contributor to increased fatigue [69], and future fatigue studies would benefit from measuring iron levels concurrent with hormone levels.

## 5. Conclusions

The results of this study suggest that estrogen concentration might play a significant role in muscle fatigability response to tDCS. High estrogen concentrations during 4 mA tDCS over the left M1 resulted in greater performance fatigability, while FI during low estrogen levels was similar in both stimulation conditions. This increased fatigability (poorer performance) might have been from cortical overexcitation which, in turn, might have increased leg muscle fatigability. Average muscle activity of the right knee extensors was also affected by tDCS condition, but not by estrogen levels. Thus, increased fatigability might stem individually from sex hormone effects at the neuromuscular level, or an increased neural drive from the combination of high estrogen and brain stimulation. Future studies should consider the variability of estrogen levels across menstrual cycles and use multiple methods to confirm that a regular cycle has occurred, in addition to tracking other serum hormones and iron levels throughout the menstrual cycle.

## Figures and Tables

**Table 1:** Estrogen levels along with common sensations and the severity of each sensation.

	Average Estrogen Level (pg/mL)	*Itching*	*Burning*	*Tingling*	*Prickling*
Active (High)	157.7 ± 101.5	(3) 4.3 ± 1.5	(3) 5.3 ± 2.1	(3) 3.3 ± 2.1	(1) 5
Active (Low)	31.9 ± 10.9	X	(3) 5.3 ± 2.3	(3) 4.7 ± 1.2	(2) 5.5 ± 2.1
Sham (High)	137.2 ± 114.0	(3) 4.5 ± 2.1	(4) 5.3 ± 2.1	(3) 4.3 ± 1.5	(4) 4.0 ± 2.6
Sham (Low)	42.9 ± 25.1	(5) 3.4 ± 1.7	(4) 4.75 ± 1.9	(2) 3.4 ± 1.7	(2) 4.0 ± 0

Average levels (pg/mL) during the high and low estrogen conditions and the most common sensations and severity reported during active and sham stimulation. Data are presented as mean ± SD. Sensations were measured on a 10-point Likert scale (i.e., 1 = almost non-existent; 10 = almost unbearable). For each sensation, the number of subjects reporting each sensation is shown in parentheses. X = no Itching reported.

## Data Availability

The data that support the findings of this report will be made available upon request to the corresponding author.
